# Advantages of ligating the rectum with gauze pad band in laparoscopic anterior resection of rectal cancer: a propensity score matched analysis

**DOI:** 10.1186/s12893-022-01822-6

**Published:** 2022-10-29

**Authors:** Yang Liu, Hengduo Qi, Chun Deng, Zhenyu Zhang, Zhi Guo, Xiaojun Li

**Affiliations:** 1 Department of General Surgery, Shaanxi Provincial People’s Hospital, Xi’an, Shaanxi China; 2 Yan’an University, Yan’an, Shaanxi China

**Keywords:** Gauze pad band, Rectal neoplasms, Laparoscopy, Propensity score matching, Rectal transection, Pathology

## Abstract

**Purpose:**

It is difficult to maintain sufficient tension throughout laparoscopic anterior resection with total mesorectal excision, which causes a decline in surgical quality. We used a soft, inexpensive gauze pad band pulling the rectal tube to analyze the effect of surgery.

**Methods:**

A gauze pad band was positioned at the proximal of the tumor, followed by fastening the rectal tube and ligating the rectum. 233 patients undergoing laparoscopic anterior resection for mid to low rectal cancer were enrolled between January 2018 and December 2020. After propensity score matching, 63 cases were selected in gauze pad band group and 126 cases were selected in traditional group. The two groups were compared in preoperative, intraoperative, and pathological characteristics.

**Results:**

Compared to traditional group, the median operation duration (203 min vs. 233 min, *p* < 0.001) and the median intraoperative bleeding (48 ml vs. 67 ml, *p* < 0.001) were lesser in gauze pad band group. A higher percentage of one cartridge transection of rectum (36/63 vs. 51/126, *p* = 0.030), shorter length of cartridges used (6.88 ± 1.27 cm vs. 7.28 ± 1.25 cm, *p* = 0.040), and longer distal resection margin (2.74 ± 0.76 cm vs. 2.16 + 0.68 cm, *p* < 0.001) were found in the gauze pad band group. The completeness of total mesorectal excision (61/63 vs. 109/126, *p* = 0.022), harvested lymph nodes (19 vs. 17, *p* < 0.001) and positive lymph nodes (1 vs. 0, *p* = 0.046) were higher in gauze pad band group.

**Conclusion:**

Ligation of the rectum with a gauze pad band allows for a reduction in operative time and intraoperative bleeding while increasing the rate of one cartridge transection. It also protected the quality of total mesorectal excision and membrane anatomy.

*Trial registration:* Not applicable.

## Introduction

Being one of the most prevalent malignancies in the world, rectal cancer has the third greatest incidence and second fatality rate [1]. The principle of total mesorectal excision (TME) has been well accepted by rectal surgeons worldwide with reduction of recurrence, improvement of disease-free and overall survival [2]. It requires a mobilization through the avascular embryologic plane to dissect the tumor and mesorectum [3].

At present, surgery has always been the main curative method used for rectal cancer and the laparoscopy has been demonstrated with similar results compared to open surgery [4]. However, laparoscopic surgeons still have anatomical, technical and visual restrictions when dissecting the rectum deep in the pelvis with rigid instruments. Owing to the limitation of the narrow pelvis for middle and low rectal cancer, the difficulty for surgeons to operate increased, especially in patients with male sex, obesity and bulky tumors. The appropriate tension of the tissue and the adequate exposure of the surgical field are the important factors to protect the completeness of mesorectum and decrease the damage to surrounding organs. How to ensure the better quality of TME and reduce the postoperative complications are challenges for most surgeons.

The membrane anatomy theory has stated that dissection along the fascial spaces may offer better preservation of blood vessels, nerves and rectal mesentery [5]. We have to mobilize along the fascial spaces with sufficient tension, while procedure is difficult in the deep pelvis with less tension [6]. A few articles have been reported pulling the rectum in laparoscopic rectal cancer surgery by tools. The number of cases in early related articles was only 10 to 25 [7–9], which was not discussed in depth. In contrast, while recent studies mainly discussed that ligation of the rectum can reduce the number of cartridges related to increased anastomotic leakage (AL) [10, 11] and improve the recent outcomes of surgery [10–12], but did not evaluate the pathological quality. In addition, because some of the ligation tools [11, 12] selected by the institute cannot be untied, so it cannot be moved into the pelvis according to the specific situation, the hard tools are easy to damage the rectal canal and destroy the integrity of the mesorectum.

In this paper, we introduce a very convenient pull method in laparoscopic anterior resection (LAR) of middle and low rectal cancer: ligate the rectum with the gauze pad band (GPB). This technique is very frequently used in southeast Asia, but no reports on its advantages have been made so far. The soft texture of this pulling material does not damage the mesentery and is convenient available at no additional cost. Unlike the previous study in the distal rectum [7–9, 11, 12], we ligate the rectal canal at the proximal end of the tumor. After propensity score matching (PSM), this study retrospectively analyzed the results of pulling the rectum in improving the intraoperative and postoperative outcomes, protecting the completion of TME and membrane anatomy.

## Methods

### Patients

Every rectal adenocarcinoma patient undergoing LAR for middle and low rectal cancer with TNM stage I, II, III between January 2018 and December 2020 was included in this study. We excluded patients with a history of preoperative abdominal surgery or who underwent emergency surgery. Patients with urinary or sexual dysfunction preoperative and those without colonoscopy and pathology reports were not included. Patients with distant metastases were excluded by Chest CT scan, abdominal CT scan and CT or MRI before surgery. Patients who had complete response after neoadjuvant chemoradiotherapy suffered a “watch and wait” nonoperative management approach and were excluded from the study. Finally, 76 patients using GPB and 157 patients using traditional method were selected in our study. All patients selected in this study signed informed consents. Baseline demographic, intraoperative, postoperative, pathological data were collected and postoperative complications were graded with respect to the Clavien-Dindo classification system [13].

### Tumor location and staging

According to ESMO guideline, the tumors were classified as low (≤ 5 cm), middle (> 5 cm, ≤ 10 cm) and high (> 10 cm, ≤ 15 cm) [14]. The distance between the tumor lower edge and the anal margin was measured by colonoscopy and digital rectal examination. We performed tumor staging and classification by preoperative colonoscopy, pelvic MRIs, and pathological results according to the 7th edition of AJCC guideline [15].

### Propensity score matching (PSM)

PSM was performed for the aim of minimizing selection bias caused by retrospective analysis [16]. Bivariate logistic regression was used to calculate the propensity scores for each patient based on the covariates of tumor location, height of the tumor and neoadjuvant chemoradiation. PSM was obtained at a 1:2 ratio between two groups. Ultimately, 63 patients who underwent LAR with GPB and 126 patients who underwent LAR with traditional instruments were enrolled and analyzed after being matched.

### Gauze pad band (GPB)

**Fig. 1:**
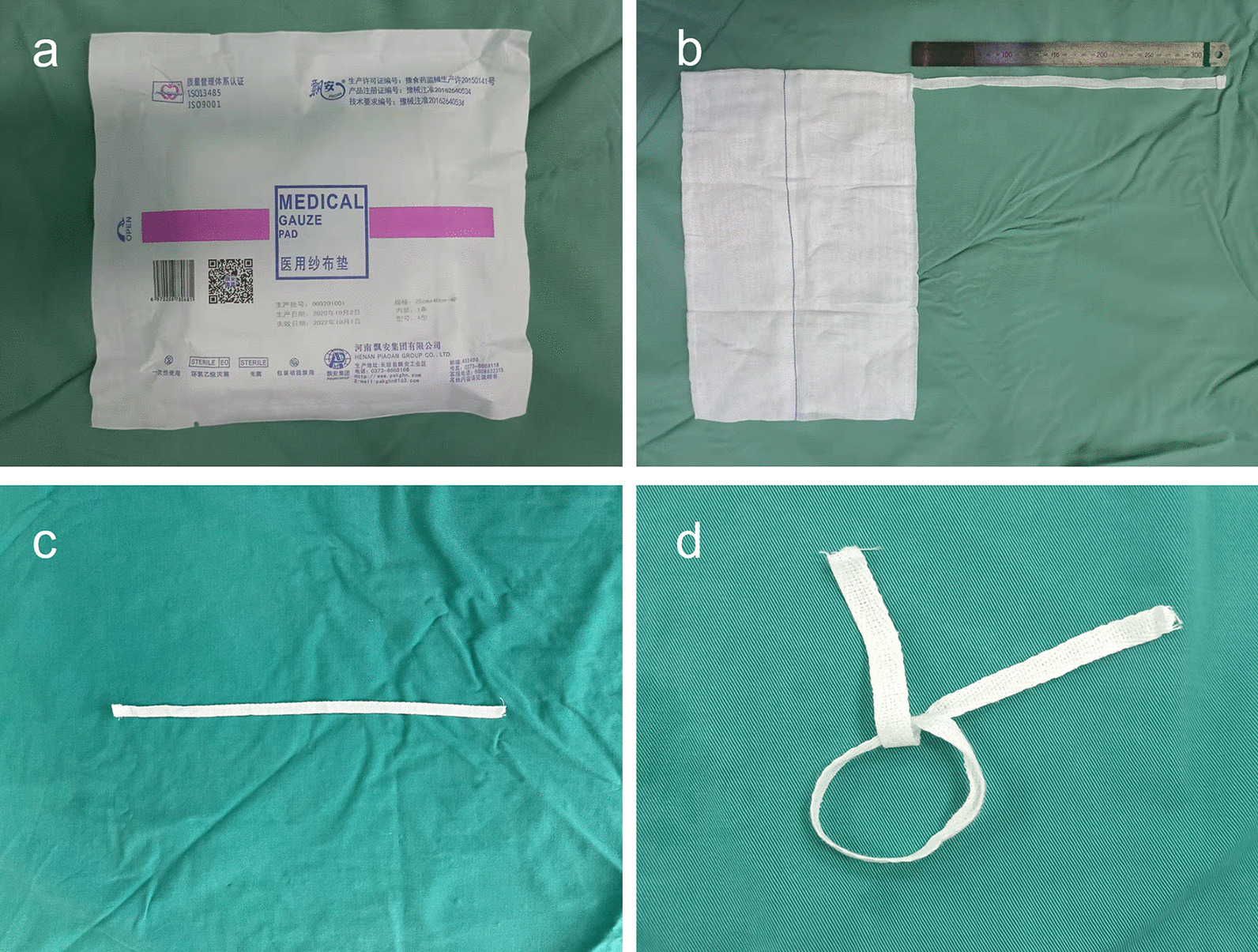
The medical gauze pad band

The GPB (Henan Piaoan Group Co., Ltd., China) used in the procedure is a tape over the medical gauze pad with a length of 33.0 cm and a width of 1.1 cm (Fig. 1), which is cut off at the attached point of gauze pad.

### Operative Technique

All surgeons who participated in the study were experts who had performed LAR for > 5 years. Four certified surgeons with over 50 LAR cases performed the operations in traditional instruments. The operations in GPB group were all performed by the same surgeon (X.L.), who had performed over 200 cases of LAR with GPB. The assistants were all qualified physicians who have participated resident training and have more than 50 cases of LAR experience, meeting the Bege’s [17] requirement of 50 procedures in the learning curve for laparoscopic rectal cancer. The assistants’ experience was classified according to the number of years after resident training (> 5 years after resident training or ≤ 5 years after resident training).

**Fig. 2:**
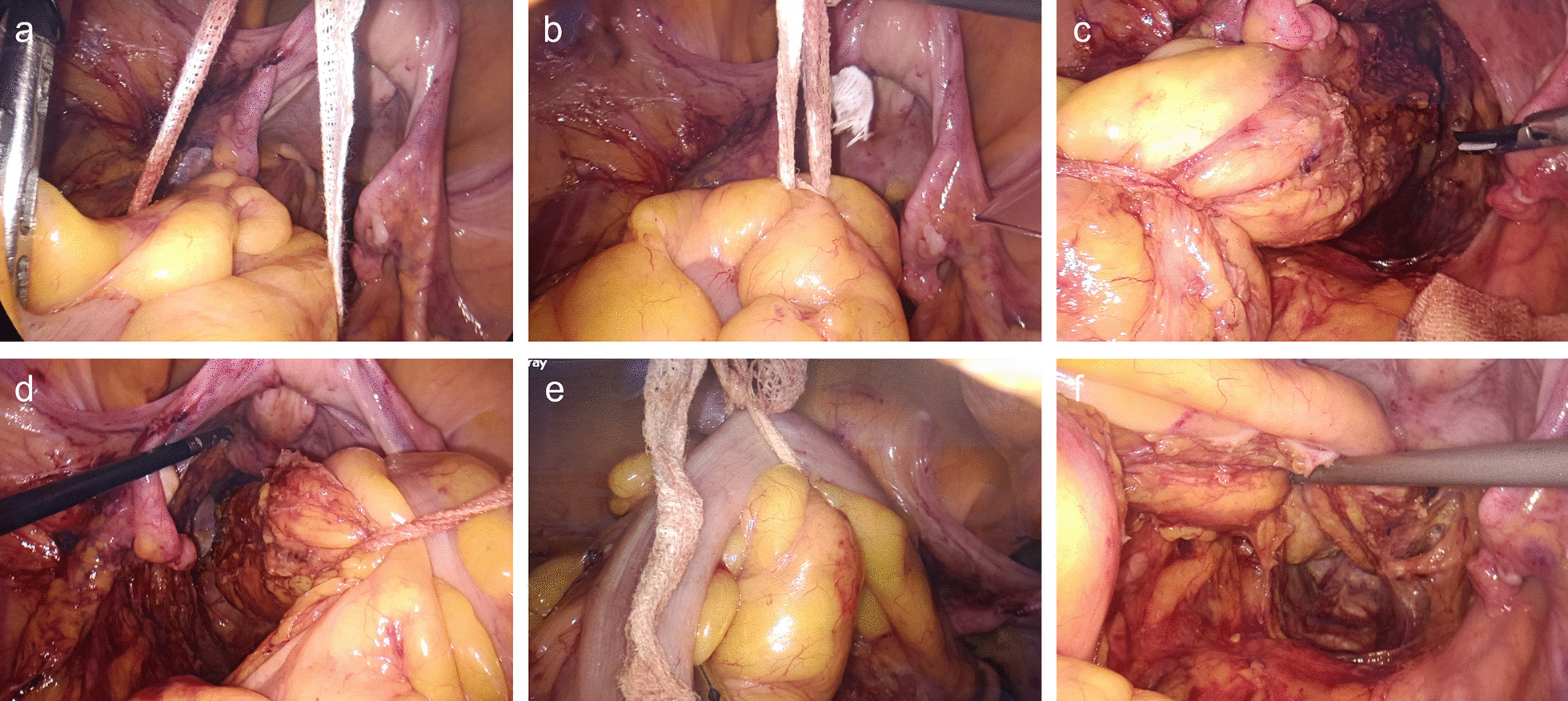
Encircle and pull the rectal canal for adequate expose of the surgical fields. **a**, **b** Encircle the proximal rectal canal and ligate with one knot. **c** Pull the rectal canal to left direction. **d** Pull the rectal canal to right direction. **e**, **f** Pull the rectal canal to abdominal direction

The operation was undertaken according to the guideline of TME [3]. When the operator mobilized the rectum and dissected it until weldeyer's fascia level, a GPB was placed into the abdominal cavity via a 12 mm trocar in the right lower quadrant and encircled the rectal canal at least 3 cm at the upper side of the tumor to ligate the rectal canal and the mesentery with only one knot (Fig. 2a, b). Assistant surgeon used instrument to grasp the knotted point and pull the rectum to provide adequate exposure when perform mesorectal dissection. The GPB will be pulled to the left, right and front side (Fig. 2c–f), exposing the corresponding surgical fields. For lower rectal cancer in the deep pelvis, the thread junction can be loosened, moved down to the appropriate site and then knotted to maintain the enough tension.

**Fig. 3:**
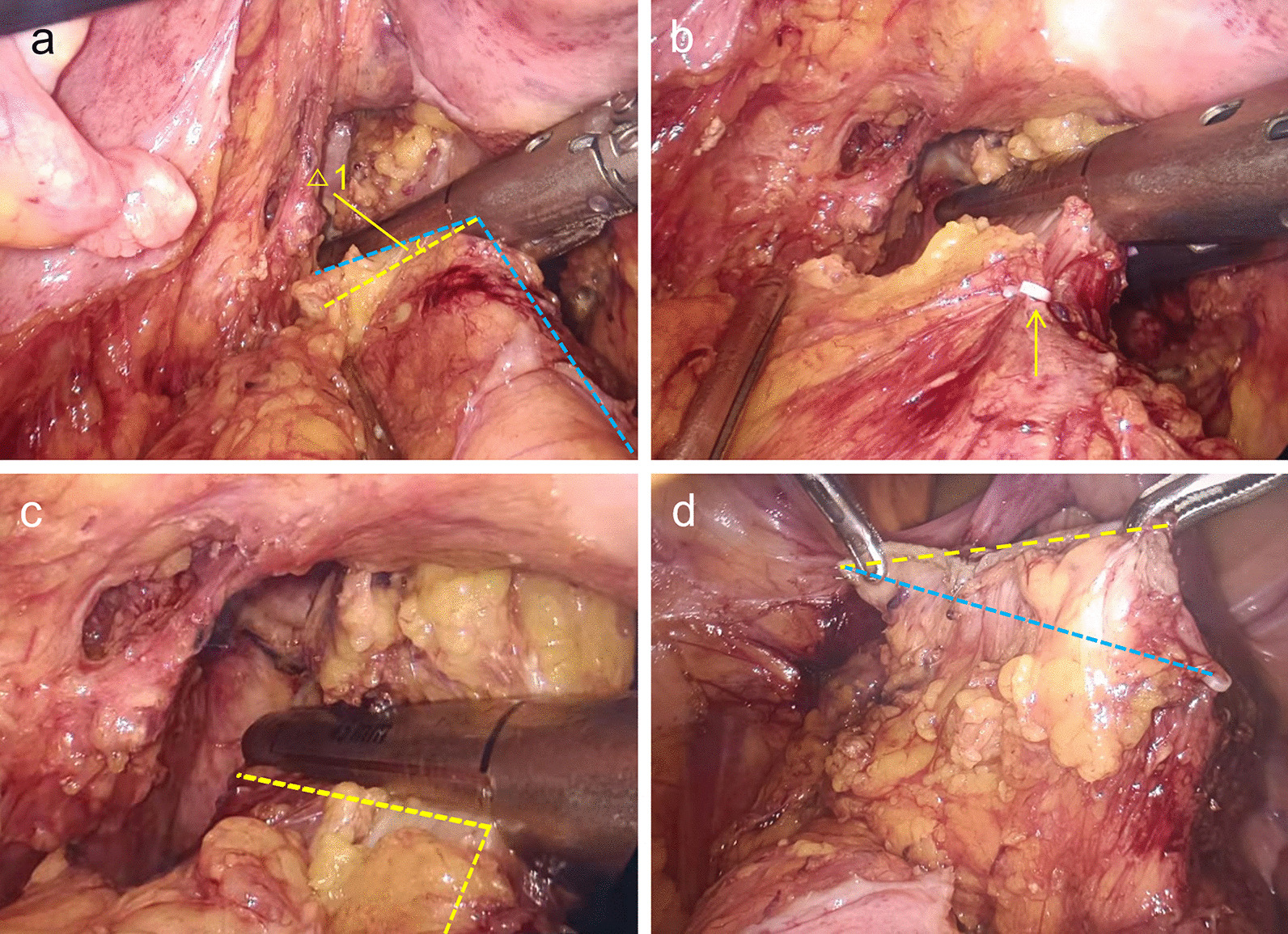
Transect the rectal canal with the traditional approach and the GPB approach. **a** The angle of inclination between the stapler and the rectum without GPB ligation. *△1* the angle between the transection line by the traditional approach and the GPB approach. **b** Marking the right edge of the rectal canal with a clip along the transection line in the traditional method. **c** The linear stapler is perpendicular to the major axis of rectal canal with the GPB to pull the rectum. **d** Without the GPB ligation, the total length of cartridges is a oblique line (blue line). After using the GPB, the total length of cartridges decreases (yellow line)

**Fig. 4:**
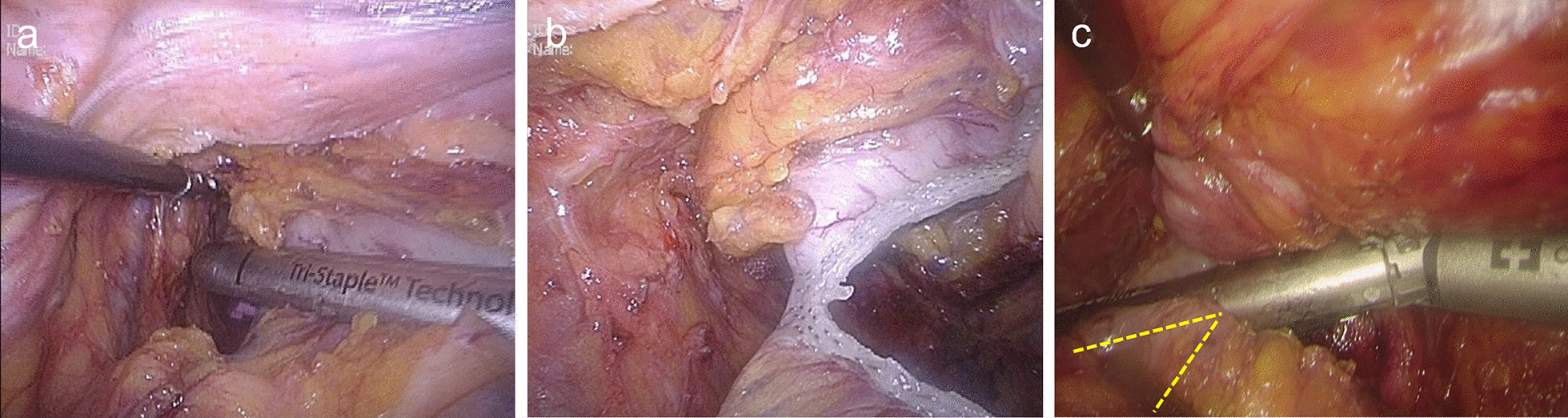
Transect the low rectum with the GPB ligation. **a**, **b** The rectum is transected with a cartridge by using the GPB to pull the rectal canal. **c** A near perpendicular angle is achieved by pulling the rectal canal when second cartridge is needed

A linear stapler (Endo GIA™ Ultra Universal stapler 60 mm or 45 mm, Covidien, USA) was positioned in the abdominal cavity for rectal transection. Using the traditional instrument to ligate the rectum, mark the transection line at the right edge of the rectal wall with a clip (Fig. 3a, b). Then use the GPB for pulling the rectum to the left side so that the fork of the linear stapler was placed at a vertical angel to complete rectal transection (Fig. 3c). The difference in the total length of cartridges with GPB and traditional instrument was showed in Fig. 3d. By pulling the rectum, the bowel was fully contacted with the stapler and the rectum could be transected with a cartridge (Fig. 4a, b). For male pelvis and obese patients, a second cartridge may be needed, but an approximate vertical angel could be achieved by pulling the rectum with GPB (Fig. 4c). Finally, the GPB was removed along with the surgical specimen from a small incision. During the operation, be careful not to tie the tumor, which may damage the integrity of the tumor and cause tumor metastasis. Traditional group used traditional instruments to clamp the rectum for surgical field exposure.

### Measurement of blood loss

The volume of blood loss was measured by negative pressure suction device and the sterile gauze pieces (Henan Piaoan Group Co., Ltd., China).

### Pathological assessment

Three physicians in our pathology department are involved in the processing of gastrointestinal surgical pathology specimens. All pathology specimens in this study were judged by two gastrointestinal pathologists according to uniform criteria, and surgeons were generally not involved in. When fewer than 12 postoperative lymph nodes (LNs) were collected in the rectal specimens, a surgeon and pathologist worked together to find the LNs.

The completeness of TME was accessed by distal resection margin, circumstance resection margin, the number of harvested LNs and positive LNs, and the integrity of mesorectum. According to Nagtegaals’ [18] research, the quality of the mesorectum was categorized in three grades: complete, nearly complete and incomplete.

### Endpoint

The primary endpoints included the assessment of the operative findings (operative time (OP), blood loss, number of cartridges used), complication rates 30 days after surgery. The secondary endpoints were the pathology reports, including the positive rate of circumferential and distal resection margin, R0 resection rate, number of harvest LNs and positive LNs, the integrity of mesorectum and the TNM stage.

### Statistical analysis

The data were analyzed by using the χ2 test for comparisons of categorical parameters. The continuous parameters were compared using Student’s *t* test or the *Mann–Whitney U* test. A *p* value < 0.05 was defined as statistically significant. The analysis was performed using SPSS version 26.0 (IBM Corp., Armonk, NY, USA).

## Results

### Postmatching baseline characteristics

Table 1 shows the baseline characteristics of the two groups after PSM. Each group consisted of 63 and 126 patients, and the two groups were well matched with no significant differences.

**Table 1:** Prematching and postmatching baseline characteristics

Characteristics	Prematching baseline characterstics		*p*	Postmatching characterstics		*p*
	GPB group (76)	NGPB group (157)		GPB group (63)	NGPB group (126)	
Male	44 (57.9%)	97 (61.8%)	0.569	37 (58.7%)	79 (62.7%)	0.597
Age, year, mean ± SD	63.95 ± 13.13	63.82 ± 12.19	0.940	63.75 ± 12.92	63.28 ± 12.73	0.814
BMI			0.697			0.522
> 25 kg/m^2^	28 (36.8%)	62 (39.5%)		21 (33.3%)	48 (38.1%)	
≤ 25 kg/m^2^	48 (63.2%)	95 (60.5%)		42 (66.7%)	78 (61.9%)	
Alb, g/L, mean ± SD	36.53 ± 5.17	35.95 ± 5.24	0.425	36.65 ± 5.14	36.18 ± 4.89	0.539
Hb, g/L, mean ± SD	121.27 ± 12.98	119.52 ± 13.26	0.344	119.53 ± 12.75	118.95 ± 13.13	0.774
Tumor size, cm, mean ± SD	3.48 ± 1.73	3.64 ± 1.57	0.496	3.65 ± 1.64	3.47 ± 1.68	0.494
Height of distal edge of the tumor, cm, mean ± SD			0.034			0.615
	4.21 ± 2.16	4.85 ± 2.14		4.26 ± 2.26	4.44 ± 2.20	
Tumor location			0.036			0.664
Low rectum (< 5 cm)	53 (69.7%)	87 (55.4%)		43 (68.3%)	82 (65.1%)	
Middle rectum (5.1–10 cm)	23 (30.3%)	70 (44.6%)		20 (31.7%)	44 (34.9%)	
ASA score			0.890			0.741
I	15 (19.7%)	34 (21.7%)		11 (17.5%)	28 (22.2%)	
II	48 (63.2%)	94 (59.9%)		42 (66.7%)	80 (63.5%)	
III	13 (17.1%)	29 (18.4%)		10 (15.8%)	18 (14.3%)	
Preoperative serum CEA, ng/ml, mean ± SD			0.734			0.792
	6.60 ± 3.17	6.44 ± 3.27		6.64 ± 3.28	6.77 ± 3.16	
Clinical stage			0.916			0.646
I	17 (22.4%)	32 (20.4%)		14 (22.2%)	21 (16.7%)	
II	23 (30.3%)	51 (32.5%)		22 (34.9%)	46 (36.5%)	
III	36 (47.3%)	74 (47.1%)		27 (42.9%)	59 (46.8%)	
Degree of histological differentiation			0.715			0.847
Well	11 (14.5%)	17 (10.8%)		7 (11.1%)	12 (9.5%)	
Moderate	49 (64.5%)	107 (68.2%)		44 (69.8%)	93 (73.8%)	
Poor + undifferentiated	16 (21.0%)	33 (21.0%)		12 (19.1%)	21 (16.7%)	
Neoadjuvant chemoradiation	54 (71.1%)	85 (54.1%)	0.014	39 (61.9%)	71 (56.3%)	0.465
Assistant's experience			0.792			0.645
> 5 years after resident training	21 (27.6%)	46 (29.3%)		16 (25.4%)	36 (28.6%)	
≤ 5 years after resident training	55 (72.4%)	111 (70.7%)		47 (74.6%)	90 (71.4%)	

*BMI* body mass index, *ASA* American Society of Anesthesiologists, *CEA* carcinoembryonic antigen, *GPB* gauze pad band, *NGPB* none gauze pad band

### Intraoperative and postoperative characteristics

The intraoperative characteristics demonstrated in Table 2 indicated a lower median OP (203 min vs. 233 min, *p* < 0.001) and intraoperative bleeding (48 ml vs. 67 ml, *p* < 0.001) in the GPB group. The GPB group had a higher rate of rectum transection with one cartridge (36/63 vs. 51/126, *p* = 0.030), especially for male (23/37 vs. 29/79, *p* = 0.010), low rectal cancer (19/43 vs. 20/82, *p* = 0.023), high BMI (14/21 vs. 19/48, *p* = 0.038) and tumor > 5 cm (8/9 vs. 9/23, *p* = 0.032). The 30-day postoperative complications were similar between the two groups (11/63 vs. 23/126, *p* = 0.893).

**Table 2:** Intraoperative and thirty-day postoperative outcomes

Characteristics	GPB group (63)	NGPB group (126)	*p*
Operative time, min, median (range)	203 (192, 218)	233 (223, 247)	< 0.001
Blood loss, ml, median (range)	48 (36, 56)	67 (58, 76)	< 0.001
Anastomotic height, cm, mean ± SD	3.17 ± 1.55	3.14 ± 1.47	0.886
Protective ileostomy	35 (55.6%)	71 (56.3%)	0.917
Splenic flexure mobilization	26 (41.3%)	48 (38.1%)	0.673
Conversion to open surgery	11 (17.5%)	26 (20.6%)	0.604
Patients with one cartridge transection	36/63 (57.1%)	51/126 (40.5%)	0.030
Male with one cartridge	23/37 (62.2%)	29/79 (36.7%)	0.010
Female with one cartridge	13/26 (50.0%)	22/47 (46.8%)	0.794
Low rectal cancer with one cartridge	19/43 (44.2%)	20/82 (24.4%)	0.023
Middle rectal cancer with one cartridge	17/20 (85.0%)	31/44 (70.5%)	0.213
BMI > 25 kg/m^2^ with one cartridge	14/21 (66.7%)	19/48 (39.6%)	0.038
BMI ≤ 25 kg/m^2^ with one cartridge	22/42 (52.4%)	32/78 (41.0%)	0.233
Tumor > 5 cm with one cartridge	8/9 (88.9%)	9/23 (39.1%)	0.032
Patients with more than two cartridges transection			
	0	0	–
No. of cartridges,median (range)	1 (1, 2)	2 (1, 2)	0.031
Postoperative Complications (< 30 days)	11 (17.5%)	23 (18.3%)	0.893
Clavien–Dindo grade			0.974
I	4 (6.3%)	9 (7.1%)	
II	3 (4.8%)	6 (4.8%)	
IIIa	3 (4.8%)	4 (3.2%)	
IIIb	1 (1.6%)	4 (3.2%)	
Types of complications			
Anatomostic leakage	2 (3.2%)	4 (3.2%)	1.000
Anastomotic bleeding	1 (1.6%)	2 (1.6%)	1.000
Hemorrhage	1 (1.6%)	3 (2.4%)	1.000
Surgery site infections	4 (6.3%)	6 (4.8%)	0.909
Urinary retention	3 (4.8%)	7 (5.6%)	1.000
Thrombosis, thrombus, or embolism	0	1 (0.8%)	1.000

*BMI* body mass index, *GPB* gauze pad band, *NGPB* none gauze pad band

### Pathological characteristics

Table 3 describes pathological outcomes after surgery. The GPB group had a longer distal resection margin (DRM) than traditional group (2.74 ± 0.76 cm vs. 2.16 + 0.68 cm, *p* < 0.001). This difference is mainly for low rectal cancer (1.78 ± 0.67 cm vs. 1.23 ± 0.59 cm, *p* < 0.001). For middle rectal cancer, the DRM was not statistically different (3.03 ± 0.83 cm vs. 2.97 ± 0.77, *p* = 0.641). The GPB group had a shorter length of cartridges (6.88 ± 1.27 cm vs. 7.28 ± 1.25, *p* = 0.040). Statistically, the GPB group had more harvested LNs (19 vs. 17, *p* < 0.001) and positive LNs (1 vs. 0, *p* = 0.046), had a great number of cases with completeness TME (61/63 vs. 109/126, *p* = 0.022) than traditional group.

**Table 3:** Pathological assessment of patients

Characteristics	GPB group (63)	NGPB group (126)	*p*
No. with circumferential margin positivity	1 (1.6%)	4 (3.2%)	0.873
No. with negative margin (≥ 1 mm)	61 (96.8%)	119 (94.4%)	0.717
R0 ressection rate	60 (95.2%)	115 (91.3%)	0.492
Distal ressection margin,cm,mean ± SD	2.74 ± 0.76	2.16 + 0.68	< 0.001
Low rectum (< 5 cm)	1.78 ± 0.67	1.23 ± 0.59	< 0.001
Middle rectum (5.1–10 cm)	3.03 ± 0.83	2.97 ± 0.77	0.641
Total mesorectal excision			0.022
Complete	55 (87.3%)	88 (69.8%)	
Nearly complete	6 (9.5%)	21 (16.7%)	
Incomplete	2 (3.2%)	17 (13.5%)	
Degree of histological differentiation			0.931
Well	6 (9.5%)	10 (7.9%)	
Moderate	46 (73.0%)	93 (73.8%)	
Poor + Undifferentiated	11 (17.5%)	23 (18.3%)	
Pathological tumor stage			0.730
pT1	6 (9.5%)	8 (6.3%)	
pT2	12 (19.0%)	24 (19.0%)	
pT3	45 (71.5%)	94 (74.7%)	
Pathological nodal status			0.019
pN0	30 (47.6%)	75 (59.5%)	
pN1	19 (30.2%)	41 (32.5%)	
pN2	14 (22.2%)	10 (8.0%)	
Stage			0.294
I	11 (17.5%)	26 (20.6%)	
II	19 (30.2%)	49 (38.9%)	
III	33 (52.3%)	51 (40.5%)	
Tumor size, cm, mean ± SD	3.65 ± 1.64	3.47 ± 1.68	0.494
No. with lymphovascular invasion	1 (1.6%)	2 (1.6%)	1.000
Total length of cartridges, cm, mean ± SD	6.88 ± 1.27	7.28 ± 1.25	0.040
No. of lymph nodes examined, median (range)	19 (17,22)	17 (14,20)	< 0.001
No. of positive lymph nodes, median (range)	1 (0,3)	0 (0,2)	0.046

*GPB* gauze pad band, *NGPB* none gauze pad band

## Discussion

With the widely development of LAR, the understanding of surgical skills, careful anatomy have also gradually deepened. Performing high quality TME, shortening OP and reducing intraoperative unintentional injuries are the goals pursued by surgeons. According to the theory of membrane anatomy, LAR with high BMI, male narrow pelvis and sphincter-preserving surgery is a huge challenge for most rectal surgeons. One of the key reasons is lack of maintain enough tension in LAR. We describe here a convenient method for encircling up the proximal rectal canal, attempt to enhance the quality of TME surgery.

Different physicians have different understandings of GPB technique, they often decide whether to use GPB based on their own preferences and their cooperation with their assistants. After performing 200 cases of LAR for middle and low rectal cancer, although our team has acquired some experience and has become more skilled, we still felt that the use of GPB could result in shorter OP and more standard TME, therefore we conducted this study. We hope that our study will generate other surgeons' interest in GPB technique and provide a theoretical basis for the advantages of using GPB.

### Perioperative characteristic

Most studies have shown that extended OP is related to increased risks of surgical site infections (SSI) [19] and AL [20]. Increased intraoperative bleeding has been reported to be associated with increased postoperative complications [21]. In the present study, less OP (203 min vs. 233 min, *p* < 0.001) and less intraoperative bleeding (48 ml vs. 67 ml, p < 0.001) were found in the GPB group, which similar to the results of Akiyo Matsumoto [10] and Sang Woo Lim [12]. The limitations of performing surgery in a narrow pelvis made it more difficult to provide adequate exposure and maintain proper tension [6]. The GPB provided sufficient tension to pull the rectum, which expose the surgical field in the deep pelvis. This allowed us to mobilize the rectum along the holy plane and avoid intraoperative damage to blood vessels, nerves and organs, which leads to shorter OP and lower blood loss [22]. In our study, the blood loss in GPB group was lesser than NGPB group, it is only few milliliters of blood, although having statistical significance, the effect on postoperative complications was not significant. Less blood loss could decrease the influence on surgical field, allowing for a clearly anatomical level, avoiding damage to the pelvic nerves and helping to reduce OP. Postoperative complication is one of the core indicators to assess postoperative recovery. The complication rate between the two group were similar (11/63 vs. 23/126, *p* = 0.893), consistent with the previous findings [10, 12], which demonstrated the use of GPB is safe and feasible. Previous studies have reported that OP is associated with SSI [19]. In this study, despite the shorter OP in the GPB group, there was no reduction in the incidence of SSI (4/63 vs. 6/126, *p* = 0.909). First, all patients in this study underwent LAR which had a reduced incidence of SSI compared to open abdominal surgery [23]. Second, we made only a small incision in the abdomen and used an incision protector for specimen removal to achieve a lower incidence of SSI [24].

### Multiple cartridges and AL

Long-term survival outcomes after rectal cancer surgery can be affected by AL [25]. Numerous studies have proposed occurrence of AL is associated with increased use of cartridges during surgery [25–27]. With this technique, we made fewer cartridges (1 (1,2) vs. 2 (1,2), *p* = 0.031) and increased the percentage of single cartridge transection (36/63 vs. 51/126, *p* = 0.030) in GPB group, the results were similar to Wang [11]. A narrow pelvis may led to an inevitable oblique transection with the linear stapler, thus increased the number of cartridges used and total length transected [28]. Repeated stapling at the same closed end [29] may cause tissue ischemia locally and lead to AL [30]. In the present study, we used GPB pulling the rectum to the cranial side to overcome the restriction of the rigid instrument in the narrow pelvis, keep the linear stapler be perpendicular to the major axis of rectal canal, shorten the total length of cartridges (6.88 ± 1.27 cm vs. 7.28 ± 1.25, *p* = 0.040) and reduce the number of cartridges used.

The AL is related with poor oncologic prognosis [31, 32]. Increased use of cartridges was a risk factor for AL [25–27, 29, 33]. The number of cartridges reduced, but the incidence of AL (2/63 vs. 4/126, *p* = 1.000) seemed not decrease in GPB group. Firstly, all patients underwent LAR in this study, the incidence of AL has decreased compared to open surgery [34, 35]. Secondly, the occurrence of AL was also influenced by non-technical factors such as gender, ASA score, preoperative (chemo)radiation therapy, intraoperative complications, precompression before stapler firings, blood supply [30, 36, 37]. A review of the 2 cases with AL in GPB group revealed that 2 patients were both male and 1 had received prolonged chemotherapy therapy preoperatively, which probably increased the incidence of AL. Thirdly, protective ileostomy can reduce the symptoms of AL [38], the protective ileostomy number of patients with a protective ileostomy was higher in both groups, which may hiding the occurrence of postoperative AL. Fourthly, only the patients with AL grade B and C were counted, which may be one of the reasons for the no statistical difference [39]. Additional researches are needed to confirm that GPB could reduce the incidence of AL.

### Pathology

The 9th edition of the JCCRC [40] stated that if tumor is located above or below the perirectal reflection, DRM needs to be at least 3 cm or 2 cm from the tumor lower edge. LAR seemed difficult to obtain adequate DRM, especially for male narrow pelvis and high BMI [6, 41, 42]. Nowadays, relevant studies have now confirmed that DRM of more than 1 cm should be a safe distance [43, 44]. In this study, the mean length of DRM was longer in GPB group (2.74 ± 0.76 cm vs. 2.16 + 0.68 cm, *p* < 0.001), especially for low rectal cancer (1.78 ± 0.67 cm vs. 1.23 ± 0.59 cm, *p* < 0.001). It seems similar to the findings of Wang et al. [11] and Akiyo Matsumoto et al. [45]. Sang Woo Lim et al. [12] have reported that DRM did not become longer, but they have discussed that adequate dissection could bring a safe DRM by pulling the rectum. The difficulties to expose the surgical field and get standardized distal margin due to the narrow pelvic space, low tumor position and tissue edema [6], with particularly reference to low-lying rectal cancer or after neoadjuvant therapy. By using a GPB to encircle the proximal rectal canal, the rectum could be pulled from the deep pelvic cavity for fully exposure of surgical field, further dissect the distal rectum and get adequate length of distal margin.

Rectal cancer surgery based on TME principles reduced local recurrence rate and improved 5-year survival rate [2]. Depending on the classification described by Nagtegaal, the integrity of TME can be categorized into complete, nearly complete, and incomplete [18]. In this study, we included the complete and nearly complete of mesentery as standard specimens. 61 (96.8%) specimens have met the standard in the GPB group while 109 (86.5%) specimens in the traditional group have done (*p* = 0.022). Surgical field exposure in the deep pelvis and the use of rigid instruments increased the difficulty of anatomy [6, 46] and decreased the quality of TME completion [47]. In this study, we minimized the restriction of the narrow pelvic structures by using GPB to encircle the rectal canal and pull the rectum to the cranial side. Unlike rigid instruments, GPB could pull the rectum into every direction in order to expose the tissue around the rectum and avoid damage the integrity of the rectal mesentery. Inadequate elevation of the rectum confused us when dissected the mesentery posteriorly. By stretching the rectum to the ventral side, the laparoscopic can further move to the deep pelvis, helping to identify the anatomical level more clearly and improving the integrity of the rectal mesentery.

The number of detected LNs guarantees the accuracy of postoperative pathological staging [48]. Our study found that the GPB group dissected more harvested LNs (19 vs. 17, *p* < 0.001) and positive LNs (1 vs. 0, *p* = 0.046). Previous studies have pointed out [49, 50] the length of resected rectal canal was associated with the number of LNs obtained. We have found that the length of DRM increased in the GPB group, we could remove a sufficient length of rectal canal to ensure completely resection of the LNs.

This study has several limitations. First, this was a single retrospective study, which may have influenced the results, although PSM was used to reduce selection bias. Second, patients in the traditional group were operated by four certified and experienced surgeons, but surgical procedures may have differed depending on surgeon preference, technique, and surgical experience. Further prospective and randomized clinical trials are needed in the future to overcome the limitations of retrospective design and selection bias.

## Conclusion

In summary, using GPB to pull the rectum could reduce the OP and intraoperative bleeding, increase the length of DPM and reduce the number of cartridges used; improve pathological results for increasing the number of complete TME and harvested LNs. Therefore, we believe that using a GPB to pull the rectum is safe and feasible for rectal cancer.

## Data Availability

The datasets used and/or analysed during the current study are available from the corresponding author on reasonable request.

## Abbreviations

TME: Total mesorectal excision
AL: Anastomotic leakage
LAR: Laparoscopic anterior resection
GPB: Gauze pad band
PSM: Propensity score matching
OP: Operative time
BMI: Body mass index
DRM: Distal resection margin
LNs: Lymph nodes
SSI: Surgical site infections
ASA: American Society of Anaesthesiologists
CEA: Carcinoembryonic antigen
